# Psoriasis Therapy and Skin Cancer: A Review

**DOI:** 10.3390/life11101109

**Published:** 2021-10-19

**Authors:** Beatriz Butrón-Bris, Esteban Daudén, Pedro Rodríguez-Jiménez

**Affiliations:** Dermatology Deparment, Hospital Universitario de la Princesa, 28007 Madrid, Spain; esteban.dauden@salud.madrid.org (E.D.); pedroantonio.rodriguez@salud.madrid.org (P.R.-J.)

**Keywords:** psoriasis, skin cancer, non-melanoma skin cancer, melanoma, phototherapy, treatment, biologics

## Abstract

Introduction: psoriasis is a chronic immune-mediated disease that is associated with several comorbidities, including an increased risk of malignancies, particularly skin cancer. A large number of studies have investigated whether psoriasis itself, psoriasis-associated comorbidities, or psoriasis treatment could lead to an increased risk of neoplasms. Methods: we reviewed the literature using the most important databases (PubMed, MEDLINE, ETHERIA). All articles pertaining to skin cancer associated with psoriasis disease and psoriasis therapy were included. In this review, we also discuss some of the potential underlying mechanisms for these associations, particularly regarding the multiple psoriasis therapies currently available, and their possible implications in higher incidences of skin cancer in these patients. Conclusion: evidence suggests that these patients might have a higher risk of cutaneous malignancies, especially for NMSC, compared with psoriasis-free patients. The reasons for this increased risk remain to be determined. However, high dose PUVA therapy, the immunosuppressive treatments used, and the comorbidities and habits frequently described in these patients seem to play a role in the pathogenesis of these tumors. Because of these facts, periodic screening for skin cancer is recommended in this population.

## 1. Introduction

Psoriasis is a chronic immune-mediated condition affecting 3–4% of the population worldwide. It is associated with several co-morbidities and carries an important psychological burden, especially noticeable in severe disease. These comorbidities include the presence of arthritis, metabolic syndrome-obesity, cardiovascular diseases, and malignancies, especially with skin cancer, but as well as with kidney, breast, lung, and colon cancers, lymphomas, etc. [1,2].

Although the chronic inflammatory state observed in psoriasis may induce pro-tumorigenic effects, treatments for psoriasis, such as ultraviolet light and systemic therapies (conventional immunosuppressants, biologics, and novel treatments with small molecules) may put patients with psoriasis at greater risk for cancer [3].

Additionally, several cytokines, such as tumor necrosis factor (TNF)-α, interleukin (IL)-1β, IL-12, IL-17A, IL-22, and IL-23, upregulate the production of reactive oxygen species and the hyperproliferative keratinocyte state seen in psoriasis, and are shown to play a role in some malignancies [1].

Furthermore, the effect of both the chronic inflammatory state and the used immunosuppressive pharmacological agents could be increased by the potential role of frequent comorbidities present in psoriasis; among them, cigarette smoking, alcohol consumption, and high sun exposure are considered the three main causes of the higher incidence of cutaneous malignancy in these patients reported in the literature [4,5].

Due to this concept, some authors have compared the incidences of skin cancer in different autoinflammatory diseases. Lumig et al. observed that there was a shorter time until the first NMSC and a higher rate of NMSC in psoriasis compared with arthritis rheumatoid patients (both ongoing TNF alpha-inhibitors), suggesting that some factors, e.g., specific inflammatory pathways, phototherapy, and psoriasis comorbidities might be important contributors for NMSC [6].

In recent decades, several new studies, systematic reviews, and meta-analyses on skin cancer risk in patients with psoriasis have been published, particularly for non-melanoma skin cancer (NMSC), with heterogeneous findings and conclusions. There are even studies that report an opposite view—a decrease in the prevalence of skin cancer in those patients. Paradisi et al. described a 16% lower probability of developing NMSC in psoriasis when compared with non-dermatological patients [7].

Our aim in this review was to discuss the potential risk of non-melanoma skin cancer and melanoma in patients with psoriasis and the potential underlying mechanisms for these associations between psoriasis and cutaneous malignancies.

## 2. Methods

An exhaustive literature search of Spanish and English publications in PubMed, Cochrane Library and EMBASE was performed from commencement to September 2021 using the predefined keywords. Preferably, systematic reviews, meta-analysis, and observational studies were selected if they analyzed the risk of melanoma and/or NMSC in psoriasis patients.

## 3. Chronic Inflammation and Increased Risk of Skin Cancer

The systemic chronic inflammation described in all autoinflammatory diseases might be related to an increased risk of cancer. A classic example extensively studied is the relation between inflammatory bowel diseases and the higher risks of certain cancers (colorectal carcinoma) [8].

Following this line of thought, it is understandable that chronic inflammation present in psoriasis could have some kind of role in the pathogenesis of skin cancer.

Psoriasis is a hyperproliferative skin disease with a predominant Th1/Th17 response, with an upregulated concentration of multitude cytokines (TNF-α, IL-1β, IL-12, IL-17A, IL-22, IL-23, interferon-γ, and IL-13). Furthermore, these changes in the inflammatory pathways in psoriasis are, in turn, associated with increased production of reactive oxygen species. Under these conditions of chronic inflammation and hyperproliferation, the development and progression of skin neoplasms might be favored [1].

## 4. Risk of NMSC and Melanoma in Psoriasis

### 4.1. NMSC

A recent meta-analysis provided solid data that patients with psoriasis have a higher risk of developing NMSC—both squamous cell carcinoma (SCC) and basal cell carcinoma (BCC), with the strongest evidence for the former—compared with the general population [9,10,11].

Peleva et al. suggested that patients receiving biologics for psoriasis will have previous exposure to classic systemic immunosuppression drugs (MTX, cyclosporine, etc.) and phototherapy, all of which might drive one’s risk of developing skin cancer. The findings have always been considered in relation to the exposure of different systemic treatments, but with a special focus on 8-methoxypsoralen-ultraviolet-A (PUVA) therapy, which has the potential to induce p53 mutations and contribute to the development of NMSC in psoriasis patients [11].

In addition, some registries have linked exposure to TNF therapies or methotrexate (MTX) to the increased risk of BCC, but not to an increased risk of SCC, which has been associated with previous exposure to PUVA and ciclosporin [10,12].

The Psoriasis Longitudinal Assessment and Registry (PSOLAR) is an observational study that included a total of 12,000 patients with psoriasis, who received ongoing phototherapy or systemic therapy without previous history of BCC and SCC, with a follow-up of 8 years. The findings showed a significantly increased risk of BCC in TNF alpha-inhibitors and the MTX group, but not for SCC [12].

Of note, the increased risk of NMSC in psoriasis seems to be independent of disease severity or the presence or absence of arthritis [13].

On the contrary, a large retrospective study reported that patients with psoriasis may have a lower risk of skin cancer. The authors postulate that cutaneous malignancies rarely develop near psoriatic plaques, suggesting a protective role of psoriasis-related pro-inflammatory cytokines against melanogenesis, melanocyte growth, and progression to nevi, focusing the attention on a high expression of p16 [11].

### 4.2. Melanoma

While the high risk for keratinocyte carcinomas is particularly strong, the link between psoriasis and malignant melanoma (MM) remains questionable because results from different studies are heterogeneous [3,13].

One retrospective study included a total of 61,692 patients with psoriasis; researchers analyzed the incidence of MM compared with matched control individuals, results showed that there was a higher risk of developing MM in psoriasis patients, especially those with severe disease, particularly notorious for in situ melanoma [14,15], whereas other studies reported similar incidences of these tumors, with respect to the general population.

Regarding the effect of the distinct treatments—no discrepancies have been found among patients with psoriasis treated with different therapies (phototherapy, systemic agents, biologics, or topical treatments).

Shamarke et al. reported an increased risk of melanoma in the ongoing biologics of patients compared with their counterparts receiving classic systemic therapy (biologic-naïve), but without statistically significant [16].

Moreover, there appears to be no decrease in survival with these immunosuppressive therapies in patients with psoriasis who develop a malignant melanoma [3,17].

## 5. Skin Lymphoma in Psoriasis

Because of its lower prevalence compared with NMSC and melanoma, the coexistence of cutaneous lymphoma and psoriasis has not been studied in depth. Although, some authors have described a higher incidence of skin lymphoma in the psoriasis population, Frentz et al. observed, in a cohort of 6910 psoriasis patients, an increased prevalence of mycosis fungoides (SIR = 15.1, 95% CI 4.1–38) at the first year of follow-up; however, these findings could be justified by the confusion among psoriasis and mycosis fungoides [18]. In this line, Gelfand et al. described a raised risk of cutaneous T-cell lymphoma in psoriasis, particularly in patients with severe disease, without being able to rule out another error in diagnosis between both entities [19].

## 6. Risk of Skin Cancer in Others Cutaneous and Not Cutaneous Autoinflammatory Diseases

Over the years, the increased risk of neoplasms in immune-mediated inflammatory diseases has been attributed to multiple factors: chronic inflammation, immunosuppressive therapy (biologics), altered specific immune pathways, etc. The evidence of an increased risk of cancers has been described for decades in multiple autoinflammatory disorders, such as rheumatoid arthritis (RA) or inflammatory bowel disease (IBD), always in special relation to a wide use of immunosuppressive therapies [19,20].

Some authors have related this risk to the introduction and use of biologics. Wang et al. referred patients—with RA ongoing TNF antagonists—had a higher risk of developing NMSC, in particularly SCC, compared with those RA patients without anti-TNF [19].

In this line, similar data have been observed with IBD, with higher incidences of non-melanoma skin cancer associated with the use of anti-TNF therapies, but also with thiopurines, showing an increased risk of squamous cell carcinoma in patients with IBD, with this therapy alone or in combination with anti-TNFs. Likewise, anti-TNFs in IBD have been associated with a risk of melanoma, particularly in Crohn’s disease [20].

In the field of cutaneous autoinflammatory pathologies, most of the research has focused on psoriasis, both because of its prevalence and because of the widespread use of immunosuppressive therapies in this disorder. That is why most of the available data have been obtained from these patients and scarce information exists about the association between skin cancer and other skin diseases, such as atopic dermatitis, where the use of immunosuppressive therapies is also very common [9,13,20,21].

Garritsen et al. investigated the occurrence of NMSC in a group of 557 patients with atopic dermatitis treated with oral immunosuppressive drugs; the results, with some limitations, showed an increased risk of SCC compared with the general Dutch population [20].

In a Danish cohort of 31,330 patients with atopic dermatitis, a lower incidence of malignant melanoma was reported, with an increased risk of SCC and BCC, but regardless of the type of therapy used. The authors suggested a possible role of the immunosuppressive agents combined with UV phototherapy to explain the link between NMSC and atopic dermatitis. At the same time, based on the inverse incidence of melanoma in this cohort, they correlated the T helper-1 cell response observed in DA with an inhibition in melanocyte proliferation and nevus formation [21].

In general, it seems evident that immunosuppressive therapy plays a fundamental role in the development of skin cancer, but the effect of the specific immune-mediated inflammatory process of the disease itself must not be overlooked.

## 7. Psoriasis Therapies and Skin Cancer

The association between systemic immunosuppressive therapies and skin cancer has been widely described for decades, not only in autoimmune pathologies, but also in other scenes, such as solid organ transplantation where these therapies have been used for a long time, at high doses, with a variable number of adverse effects, including an increased risk of some neoplasms, which has been extensively studied in this population [22].

In contrast to what happens in these patients, the doses and intervals used in patients with dermatological autoinflammatory diseases are much lower and the introduction of the new targeted immunosuppressive drugs seems to clearly modify the incidence of these cancers [1].

### 7.1. Chronic Use of Topical and Oral Corticosteroids

It is generally accepted that the risk for cutaneous malignancy from topical agents used in psoriasis disease is low. Studies investigated photocarcinogenicity of topical corticosteroids and vitamin D analogues have shown no increased risk of skin cancer with a high safety profile [23,24,25].

Jensen et al. found a positive association among use of oral glucocorticoids and the risk of developing NMSC, significant for basal cell carcinoma; the study had some limitations, e.g., not regarding several confounding factors, such as skin phenotype or chronic sun exposure [26].

Similar data obtained by Sorensen et al. observed a higher incidence of BCC and SCC too, in users of oral systemic glucocorticoids, with an increased risk according to doses (the higher the number of the prescriptions, the higher the risk) [27].

### 7.2. UVB Therapy (PUVA, NBUVB, and BBUVB)

Cutaneous malignancies are the most investigated cancers as a consequence of treatment with 8-methoxypsoralen plus ultraviolet A (PUVA) or a narrow band (311 nm) ultraviolet B (NB-UVB). Although UVB is a well-known carcinogen, there is no evidence for an association of UVB treatment and an increased risk of developing skin cancers. Patients receiving PUVA treatments do appear to be at an increased risk of skin cancer in a dose dependent manner, being as PUVA is associated with abnormal lentiginous melanocytic proliferations and p53 mutations contribute to the development of melanoma and NMSC [1,7,28].

Data from a US PUVA study, with about 30 years of follow-up, clearly associated PUVA therapy with skin cancer, mainly SCC. In this study, the incidence of SCC in psoriasis patients was 32 times higher than that expected for an age- and gender-matched general population. Non-exposed skin also developed SCC, including invasive penile SCC. The risk of developing tumors was shown to increase in a linear mode with the cumulative PUVA dose and persisted after treatment cessation [10].

The safe maximum number of sessions of PUVA and NB-UVB, and the safe maximum cumulative dose during a person’s lifetime, are not yet clear; some studies reported that more than 250 sessions of PUVA increased the risk of developing malignant melanoma, while patients who received more than 350 NB-UVB sessions had an increased risk of NMSC compared with those who received less than 350 [1,29,30]. However, these findings are not so evident in cohorts with phototypes III and IV [31,32]

On the other hand, the data available from UVB phototherapy have shown no increase in skin cancer, especially with <100 treatments. A modest increase in SCC has been observed only in patients treated with PUVA followed by a second treatment with broadband UVB (>300 treatments) [33].

Recent reviews show a clear discordance between European and US studies, showing a stronger association between UVB therapy and skin cancer risk in the USA, most pronounced for squamous cell carcinomas. This difference may be explained by high UVA dose exposure and the lighter phototypes in the US population [34].

In short, it seems that the benefits may exceed the risks for patients with psoriasis to choose this therapy option, due to the stronger association found with PUVA therapy, even after treatment is stopped [32]. Regarding NB-UVB therapy, large prospective studies are needed to assess its long-term safety.

Additionally, most psoriasis patients improve their skin disease during sunny periods in response to sun exposure. Accordingly, home UVB treatments and sun-seeking behavior patterns can lead to an increased risk of NMSC. High sun exposure is considered to be a risk factor for skin cancer and must be taken as a confounding factor in this population.

### 7.3. Classic Systemic Drugs (MTX, Cyclosporine, Acitretin, Fumaric Acid Esters)

Regarding oral systemic treatment in psoriasis, methotrexate is probably the most prescribed drug; however, the risk of developing skin cancer due to this treatment is uncertain.

In the observational study from PSOLAR, an increased risk of BCC (but not SCC) could be observed in patients with psoriasis treated with MTX when compared with the non-MTX treated groups, and patients not treated with biologics [12].

Moreover, other studies have associated MTX treated with an enhanced risk for both BCC and SCC in patients with psoriatic arthritis and rheumatoid arthritis. Furthermore, in a cohort of patients with rheumatoid arthritis, long-term MTX use (more than a year) significantly elevated the risk for a second non-melanoma skin cancer [17,35].

Additionally, the risk of developing NMSC, in particularly SCC, could be increased in patients treated with both PUVA and MTX [26].

On the other hand, a study suggested, in patients with personal history of MM, treatment with MTX does not raise the risk of developing a consecutive melanoma [36].

Regarding cyclosporine (CsA)—it has been studied in detail in organ transplant research, showing an increased risk for NMSC, especially SCC, in transplant patients treated with this immunosuppressant drug. However, in psoriasis, the risk of skin cancer appears to be limited, as CsA in those patients, is administered at lower doses and for shorter periods [13].

In a retrospective cohort study of 272 patients who received cyclosporine for at least 1 month (mean treatment period of 8 months), and were followed-up for 10.9 years, treatment did not elevate the risk of skin malignancies [37].

Similar to MTX, the combination of cyclosporine and PUVA in the treatment of psoriasis leads to an increase in the development NMSC (in this case, in both BCC and SCC) [38,39]. Marcil et al. reported in a PUVA follow-up study that there is a threefold higher risk for SCC in ciclosporin users than in non-users [40].

Finally, the data on the risk of skin cancers in patients treated with acitretin are limited. Nevertheless, some studies have suggested the use of oral acitretin appears to be associated with a reduction of SCC risk. Similar results have been reported in the literature for treatment with fumaric acid esters, although anecdotal reports have described increased development of malignant melanoma in patients with psoriasis with this treatment [38,41].

### 7.4. Biologic Treatment (TNFi, Anti-IL12/23, Anti-IL17A/IL17R, Anti-IL23)

Biological therapy in psoriasis has revolutionized the management of this disease. There are currently four groups of these drugs available on the market: (a) tumor necrosis factor inhibitors (TNFi) (etanercept, infliximab, adalimumab, and certolizumab), (b) interleukin IL-12/23 antagonists (ustekinumab), (c) IL-17 A inhibitors (secukinumab and ixekizumab), and IL-17 receptor antagonists (brodalumab) (d) anti-IL-23 agents (tildrakizumab, guselkumab, and risankizumab).

These therapies have important effects on innate and adaptive immune pathways, which may suppose relevant changes into cancer immunosurveillance mechanisms, especially TNFi, which predominantly target IL-1 and IL-6 in cancer pathways. As described above, the risk of NMSC and melanoma associated to TNF-α antagonists, such as etanercept, infliximab, and adalimumab has been largely studied in patients with rheumatoid arthritis and Crohn’s disease, and is not established in patients with psoriasis [11].

The data available for TNFi therapies in psoriasis have reported a higher risk of NMSC, driven by a predominant increase in SCC, compared with those patients receiving TNFi for rheumatoid arthritis or with the general population [6,42,43].

On the other hand, regarding melanoma and biologics, reports are scarce and contradictory; Haynes et al. suggested no risk for melanoma in patients with rheumatoid arthritis, inflammatory bowel disease, or psoriasis treated with TNFi, whereas Raaschou et al. observed a heightened risk for melanoma in these patients [44,45].

Similarly, one study has specifically evaluated the risk of melanoma in those receiving ustekinumab, which found a similar incidence of these tumors compared with the general population [46].

In accordance with these findings, a recent systematic review and meta-analysis suggests no differences exist for induction of melanoma between treatment of chronic cutaneous diseases with TNFi or ustekinumab and treatment with conventional systemic immunosuppressive drugs, while data on NMSC are more controversial. These results are in agreement with those obtained from the German Psoriasis Registry PsoBest, performed in 2008, with a follow-up period of 10 years; in this cohort, no relevant differences were found among any of these drugs with respect to melanoma skin cancer and non-melanoma skin cancer [47,48].

Currently, there are no reported data assessing the incidence of skin cancer in patients with inflammatory cutaneous diseases treated with biologics that selectively target IL-23 or IL-17, such as secukinumab, ixekizumab, brodalumab, tildrakizumab, guselkumab, or risankizumab.

Therefore, studies in real world practice with longer monitoring periods are required to obtain a more valuable understanding of the effects of these biologic therapies on skin cancer development.

### 7.5. New Therapies in Psoriasis (JAK-Inhibitors, Apremilast)

There is limited information in regard to the risk of skin cancer associated with the novel chemically synthetized drugs used for psoriasis treatment: apremilast, an anti-PDE4 small molecule, and tofacitinib, a Janus kinase inhibitor. Only one study analyzed their effects, finding no significant differences between these treatments and other biologics or systematic therapies [49].

The development and introduction of these targeted drugs will require further observational studies to provide more information about the risk of skin cancer with these newest therapies.

## 8. Conclusions

To date, the data provided by the literature on the risk of skin cancer in patients with psoriasis and the involvement of the different therapies used in this disease are still unclear. Data are summarized in Table 1.

It seems evident that there is an increased risk of NMSC associated with PUVA therapy and prolonged exposure to this treatment is higher when an immunosuppressive drug is associated (MTX and cyclosporine). However, regarding other treatments used for psoriasis, the information is limited, and has been obtained from studies with short follow-up times and heterogeneous populations, where relevant confounding factors were overlooked, and skin cancer incidence was not one of the main variables under study.

Several factors have contributed to complicate these studies. The presence of confounding factors in these patients (smoking habits, metabolic syndrome-obesity, prolonged exposure to the sun in sunny periods, severity disease, and the presence of inflammatory arthritis, among others) and demographic data, such as phototype and family and personal history of skin cancer, should be considered.

Studies that have analyzed the association between psoriasis treatments and malignant skin lesions are characterized by a great heterogeneity of the samples, with multiple combination therapies, different degrees of psoriasis severity, etc.

Considering all of the available information, it seems logical to assume a heightened risk in those patients with severe disease, associated or not with arthritis, as this implies more inflammation, the accumulation of systemic immunosuppressive therapies over the years, and the coexistence of several comorbidities, resulting in a significant alteration of the immune system.

In addition, results may be biased by the fact that psoriasis patients are usually followed up with by dermatology specialists, thereby increasing the possibility of an earlier diagnosis of malignant skin lesions compared with the general population without a diagnosis of skin pathology.

Furthermore, an additional factor to be considered is that the increased risk of cancer has been, per se, attributed to this pathology over the years. This effect is suggested to be based on an altered immune status resulting from the chronic inflammation of and the pathways specifically activated in psoriasis, compared to other inflammatory processes, as previously discussed with rheumatoid arthritis or inflammatory bowel disease.

New prospective studies with long-term follow-ups, homogeneous samples, and a thorough selection of the confounding factors could shed light on the increased risk of skin cancer in patients with psoriasis, clarifying the role played in carcinogenesis by the disease itself, the different associated comorbidities and the multiple therapies used.

## Figures and Tables

**Table 1:** Studies assessing the risk of developing any NMSC, BCC, and melanoma in patients with psoriasis treated with different therapies.

Therapies	Reference		Number of Patients	Cancer Risk Estimated	
				NMSC	Melanoma
PUVA (>200)	Stern et al. 2001 [50] (>200 PUVA treatments)		1380	-	IRR = 8.4; 95% (3.4–17.3) Increased risk of melanoma in patients treated with PUVA
	Stern et al. 2012 [29] (>350 PUVA treatments)		1380	SCC: IRR = 6.01, 95% (4.41–8.20) BCC: IRR = 3.09, 95% (2.36–4.06) Exposure to more than 350 PUVA treatments greatly increases the risk of SCC	
	Hearn et al. 2008 [51] (>100 PUVA treatments)		3867 (24,753 PY)	SCC: IRR 2.06; 95% (0.89–4.73) BCC: IRR 1.66, 95% (0.24–9.80)	CMM: IRR = 4.43; 95% (0.69–48.99)
				No significant association among NB-UVB treatment and BCC, SCC, or melanoma	
NBUVB	Man et al. 2005 [52]		1908	SCC: SRR = 149; 95% (18–539); *p* > 0.05. BCC: SRR = 213; 95% (102–391); *p* < 0.05) No increased risk of SCC in patients treated with NBUVB compared with general Scottish population A small but significant increase of BCC.	CMM: SRR = 187 95% (23–675) No increased risk of melanoma in patients treated with NBUVB compared to the general Scottish population
	Hearn et al. 2008 [51]		3867 (24,753 PY)	SCC: IRR 2.04 95% (0.17–17.82) BCC: IRR 1.22 95% (0.28–4.25)	CMM: IRR = 1,02 95% (0.019–12.73)
				No association was found between NB-UVB exposure alone (>100 NB-UVB treatments) (without PUVA) and any skin cancer. For NB-UVB and PUVA treated patients, there was an association with BCC, with 27 BCCs found, compared to 14.1 expected in the matched population	
	Maren W. et al. 2004 [53]		126 (726 PY)	-	No evidence for increased skin cancer risk for patients treated with NBUVB phototherapy
MTX	Stern et al. 1997 [29]		80 patients with NMSC and 297 matched controls	-	RR = 1.2 (upper bound 95% confidence interval = 1.9) MTX does not increase the risk of cutaneous malignancy
	Buchbinder et al. 2008 [54]		459 (4145 PY)	-	SIR = 3.0, 95% (1.2–6.2). Compared with the general population, patients with RA treated with MTX have an increased incidence of melanoma
	S. Polesie et al. 2020 [17]		395 patients with psoriasis who had previously been cancer-free and had a first CMM	-	OR = 1.0, 95% (0.8–13). No risk of CMM
CsA	Paul et al. 2003 [38]		1252 (PY 4377)	BCC: IR = 1.1/1000 PY; 95% (0.4–2.6). SCC: IR = 1.2/1000 PY; 95% (1.9–5.6). Increased risk of NMSC associated with cyclosporine treatment, mostly SCC	IR = 0.5/1000 PY; 95% (0.1–1.6). No risk of CMM
Adalimumab	Leonardi et al. 2011 [43]		3727 (5429.9 PY)	BCC: SIR = 1.24; 95% (0.8–1.83) SCC: SMR = 3.03; 95% (1.61–5.17). No risk of NMSC associated with adalimumab treatment	-
Etanercept	Pariser et al. 2012 [55]		4410 (4775.1 PY)	BCC: SIR: 0.55; 95% (0.37–0.80) SCC: SIR: 1.78; 95% (1.11–2.69). SIR for NMSC did not achieve statistical significance	-
Biologics combined	Asgari et al. 2017 [56]		2285 (9211 PY)	BCC: aHR = 1.23 (0.91–1.66) SCC: aHR = 1.81; 95% (1.23–2.67)	-
	Mason et al. 2018 [57]		5672 (20558 PY)	BCC: aHR = 0.84; 95% (0.45–1.54) SCC: aHR = 1.20; 95% (0.57–2.50)	
	deShazo et al. (Psolar) [12]	TNFi	TNFi + ustekinumab: 7955 Increased risk of NMSC in patients with biologics therapies.	BCC: aHR = 2.54 (1.08–5.98) SCC: aHR = 0.91; 95% (0.41.95)	-
		Ustekinumab		BCC: aHR = 1.35; 95% (0.49–3.67) SCC: aHR = 0.30; 95% (0.10–0.90)	
Tofacitinib	Burmester et al. 2020 [49]		783 (776 PY)	NMSC: IR = 0.5; 95% (0.1–1.3) No increased risk of NMSC associated with tofacitinib	-

Abbreviations: NMSC: non-melanoma skin cancer; BCC: basal cell carcinoma; SCC: squamous cell carcinoma; TNFi: tumor necrosis factor-alpha inhibitors; PY: patient years; IRR: incidence rate ratio; SRR: standardized rate ratio; OR: Odd ratio; SIR: standardized incidence rate; aHR: adjusted hazard ratio; SMR: standardized mortality rate.
